# 

**Published:** 1883-01

**Authors:** 


### BOOKS AND PAMPHLETS RECEIVED.

ANNUAL ANNNOUCEMENT ONTARIO VETERINARY COLLEGE. 1882-1883. Also names of graduates who hold the Diploma of the Council of the Agricultural and Art Association.

CATALOGUE AND AWARD OF THE DAIRY SHOW, held by the British Dairy Farmers' Association, London.

NEUF. CAS DE GUERISON DE LA RAGE par M. E. Decroix, Veterinaire principal en retraite. 8vo. pp. 32. Paris. 1882.

JOHNS HOPKINS UNIVERSITY CIRCULARS. 1882.

ANNALES ET BULLETIN DE LA SOCIETE DE MEDICINE DE GAND. 1882,

THE DISSEMINATION OF TEXAS FEVER OF CATTLE AND HOW TO CONTROL IT. pp. 14. Washington, 1882.

JOURNALS.—The Veterinary Journal, London. Der Zoologische Garten, Frankfort. Wochenschrift fur Thierheilkunde und Viehzucht, Augsburg. Schweizerisches Archiv fur Thierheilkunde und Thierzucht, Bern· Archiv fur Wissenschaftliche und Practische Thierheilkunde, Berlin. Journal de la Societe contre L'Abus du Tabac, Paris. Kansas City Review of Science and Industry. Chicago Medical Journal and Examiner. The Medical Record, New York. The College and Clinical Record, Philadelphia. Annals of Anatomy and Surgery, Brooklyn. New England Medical Monthly, Newton. Chicago Medical Review. St. Louis Clinical Record. Virginia Medical Monthly, Richmond. Nashville Journal of Medicine and Surgery. The Southern Clinic, Richmond. The Western Medical Reporter, Chicago. Journal of Cutaneous and Venereal Diseases, issued monthly, edited by H. G. Piffard, A.M., M.D., and P. A. Morrow, A.B., M.D., New York. The Blacksmith and Wheelwright, New York. National Live Stock Journal, Chicago. The Breeder's Gazette, Chicago. Cultivator and Country Gentleman, Albany. Indiana Farmer, Indianapolis. Mirror and Farmer, Manchester. Truth, San Francisco. Weekly Drover's Journal, Chicago. Agricultural Review and Journal of the American Agricultural Association, New York.

